# Electronic patient record and its effects on social aspects of interprofessional collaboration and clinical workflows in hospitals (eCoCo): a mixed methods study protocol

**DOI:** 10.1186/s12913-021-06377-5

**Published:** 2021-04-23

**Authors:** Marina Beckmann, Kerstin Dittmer, Julia Jaschke, Ute Karbach, Juliane Köberlein-Neu, Maya Nocon, Carsten Rusniok, Florian Wurster, Holger Pfaff

**Affiliations:** 1 Institute of Medical Sociology Health Services Research, and Rehabilitation Science (IMVR), Faculty of Human Sciences, Faculty of Medicine and University Hospital Cologne, University of Cologne, Eupener Str. 129, 50933 Cologne, Germany; 2 Center for Health Economics and Health Services Research, University of Wuppertal, Wuppertal, Germany; 3 Sociology in Rehabilitation, Faculty of Rehabilitation Sciences, Technical University Dortmund, Dortmund, Germany

**Keywords:** Electronic patient record, EPR, Hospital, Clinical communication, Interprofessional collaboration, Mixed methods, Digitalization

## Abstract

**Background:**

The need for and usage of electronic patient records within hospitals has steadily increased over the last decade for economic reasons as well as the proceeding digitalization. While there are numerous benefits from this system, the potential risks of using electronic patient records for hospitals, patients and healthcare professionals must also be discussed. There is a lack in research, particularly regarding effects on healthcare professionals and their daily work in health services. The study *eCoCo* aims to gain insight into changes in interprofessional collaboration and clinical workflows resulting from introducing electronic patient records.

**Methods:**

*eCoCo* is a multi-center case study integrating mixed methods from qualitative and quantitative social research. The case studies include three hospitals that undergo the process of introducing electronic patient records. Data are collected before and after the introduction of electronic patient records using participant observation, interviews, focus groups, time measurement, patient and employee questionnaires and a questionnaire to measure the level of digitalization. Furthermore, documents (patient records) as well as structural and administrative data are gathered. To analyze the interprofessional collaboration qualitative network analyses, reconstructive-hermeneutic analyses and document analyses are conducted. The workflow analyses, patient and employee assessment analyses and classification within the clinical adoption meta-model are conducted to provide insights into clinical workflows.

**Discussion:**

This study will be the first to investigate the effects of introducing electronic patient records on interprofessional collaboration and clinical workflows from the perspective of healthcare professionals. Thereby, it will consider patients’ safety, legal and ethical concerns and quality of care. The results will help to understand the organization and thereby improve the performance of health services working with electronic patient records.

**Trial registration:**

The study was registered at the German clinical trials register (DRKS00023343, Pre-Results) on November 17, 2020.

## Background

Digitalization in hospitals and across the entire healthcare sector is a significant and widely discussed topic within health policy. International comparison of digital health strategies performed by the Bertelsmann Stiftung [1] showed that in Europe, Estonia, Spain and U.K. are digitally advanced countries in terms of policy activity (e.g., state funding), digital health readiness (e.g., electronic exchange of health data) and actual use (e.g., high level of electronic health record uptake). However, in Germany, the Digital Health Index indicates the need for improvement as Germany is 16th out of 17 indexed countries [1]. Only 25.6 % of German clinics have a fully functional electronic patient record system [2]. This need for improvement is recognized in health policy [3, 4], and legislative measures to support the use of digitalization are continuously being developed (Digital Healthcare Act [Digitale-Versorgung-Gesetz], Patient Data Protection Act [Patientendaten-Schutzgesetz]). Electronic patient records (EPR) [elektronische Patientenakte, EPA]^2^) [6] enable the storage, retrieval and modification of health data using digital means instead of paper-based recording systems within one healthcare organization (here: hospital, also called in-house EPR [interne EPA]) [2, 7]. This in-house EPR is the prerequisite for compiling electronic records that can be accessed by all healthcare organizations and by patients themselves.

Cross-organizational electronic records improve the overall quality of health services delivered to patients, particularly by enabling users to think more broadly and to communicate effectively [8]. However, even within a single healthcare organization, the use of EPR may result in quality improvement. Advantages (e.g., economic benefits) brought about due to efficiency increases, such as savings in drug expenditures, improved utilization of radiology tests, enhanced charge capture, and fewer billing errors, are frequently touted in connection with the introduction of EPR [9–11]. Furthermore, when properly implemented, EPR can improve the quality of healthcare [12], increase guideline compliance and reduce medication errors [10]. Likewise, the availability of data such as real-time patient data as well as easier access to information [13] are also emphasized as benefits [14] and could result in increased patient’s safety [12]. Mohsin-Shaikh et al. [15] noted improved readability, the possibility of remote access and reduced time for certain tasks, while Saranto and Kinnunen [16] demonstrated that the standardization from EPR produces more positive than negative effects concerning the documentation quality of nurses.

However, the economic effects of EPR are still partially in doubt [10], and not all parties involved are convinced of the benefits. Safety and privacy concerns are one of the most frequently mentioned obstacles [8, 17, 18]. Middleton and colleagues [19] stated that new security risks for patients may arise due to default values within the EPR, which could, for example, lead to errors in medication orders [20]. In a study by Bani Issa [18], nurses reported feeling concerned about unauthorized usage of patient data as well as administrative security risks (e.g., improper use of data for research purposes). Furthermore, healthcare professionals are not only concerned about safety [18] but also often undergo inadequate training on how to use EPR [12, 13] and lack technical support [21]. Rathert et al. [13] observed the challenge of insufficient software interoperability even between different wards within the same hospital.

Focusing on healthcare professionals’ daily work, researchers have stated that some processes seem to be more time-consuming when using the EPR system [22], resulting in fewer possibilities for team-wide consultations [15]. In addition, several studies have identified the potentially increased workload due to the introduction of EPR [13, 15, 23, 18, 22]. For example, Pelland and colleagues [23] measured a decrease in patient-physician interaction caused by an increase in time on the computer, from physicians’ perspective. Bani Issa [18] explained the decreased patient-physician interaction by increased documentation time, from patients’ perspective. Several studies have described a reduction in direct interprofessional communication after the introduction of EPR [22, 24, 25]. In contrast, Chao et al. [26] showed increased frequency of interprofessional communication, preserving the common ground (same knowledge ground) and intraprofessional communication patterns.

The EPR system is not merely used as a documentation tool but also as a communication and collaboration tool [25]. Following Habermas [27], it is important to differentiate between communicative action and discourse. A statement corresponds with communicative action when it is understandable and ‘real’. When clarification is needed, discourse is required to solve confusion. Without EPR, synchronous [28] and personal communication in hospitals is predominantly conducted either face-to-face or via telephone to promptly clarify uncertainties and unanswered questions [29]. This communication process can lead to frequent disruptions in work activities as it often takes place ad hoc and not within the framework of regular discussion, for example during rounds [29]. The EPR system has the potential to facilitate communicative action and reduce these disruptions by enabling asynchronous communication [30, 31]. As such, EPR may better support collaboration and interprofessional communication [31]. However, effective communication requires common ground [32, 33, 26], which could be threatened by asynchronous communication [34]. The effect of EPR on collaborative communication is still uncertain [34]. Indeed, Daum [35] criticized the lack of research on the consequences of EPR for healthcare professionals.

It is thus reasonable to expect that collaboration between professionals across hospital wards changes significantly due to the introduction of EPR. This study counteracts the research gap on what these changes are by addressing the social consequences of introducing EPR. The first research question is.

A. Does the introduction of EPR lead to a change in the social aspects of interprofessional collaboration? If so, how and why?

This research question is divided into three sub-questions:

1. With whom and how do healthcare professionals communicate? How do the networks of interprofessional collaboration change with the introduction of EPR?
2. What changes in interprofessional collaboration and communication occur for healthcare professionals when working with EPR, and how do they deal with these changes?
3. What is the difference in documentation between paper-based records and EPR with regard to content and quality?

As described, changes to clinical workflow and working habits as a consequence of introducing EPR were also observed [15, 21, 36, 37]. Researchers have often attributed negative changes to a lack of knowledge on the side of clinical management and software developers about best practices for workflows [37]. Lack of knowledge results from interprofessional ignorance with regard to the workflows of different professional groups (e.g., physicians, nurses, administration) [24]. Due to this ignorance, there is the possibility that the processes on which the software is based are not fitted to the actual workflow within the hospital wards; this leads to so-called ‘workarounds’ [13, 20], such as additionally administered paper documentation [22], as well as clinical frustration, potentially resulting in patient harm [37]. On the other hand, researchers have identified optimization potential for given processes and the clinical pathway overall because of digitalization (e.g., [22, 38, 39]). The challenge is to manage digitalization and its consequences at the organizational and individual levels in such a way that an effective and people-oriented use of digitalization with minimal side effects is created [40, 41]. The clinical adoption meta-model (CAMM) provides a framework to describe workflows and challenges with clinical adoption when applying health information technology (IT) systems [42]. The model is based on various established general adoption models such as the technology acceptance model [43] and the diffusion of innovation theory [44]. The CAMM describes four dimensions – availability, use, behavior and outcome – which evolve over time and are interdependent [42]. Classification into those dimensions helps to analyze workflow at different phases of IT systems’ (in this case, EPR) adoption. Furthermore, according to the CAMM, results of the introduction of the EPR can only be measured once the last dimension (outcome) has been reached. Once this dimension is reached, five aspects of outcomes can be measured: organizational outcomes, provider outcomes, patient outcomes, population outcomes and cost outcomes.

The second aim of the study is to describe the changes in hospital work environment that occur as a result of switching from paper-based patient records to EPR:

B. Does the introduction of EPR lead to a change in clinical workflows with regard to central dimensions such as total workflow time or number of process steps? If so, how and why?

This research question is also divided into three sub-questions:

1. What is the workflow of the use case, and which changes occur because of EPR?

B2 Do safety, ethical concerns and healthcare professionals’ and patients’ experiences of clinical workflow change because of EPR (including provider and patient outcomes)?

The final sub-question aims to gain further understanding of the workflow:

B3 What is the classification level within the CAMM before and after introducing the EPR?

## Methods and analysis

### Study design

**Fig. 1:**
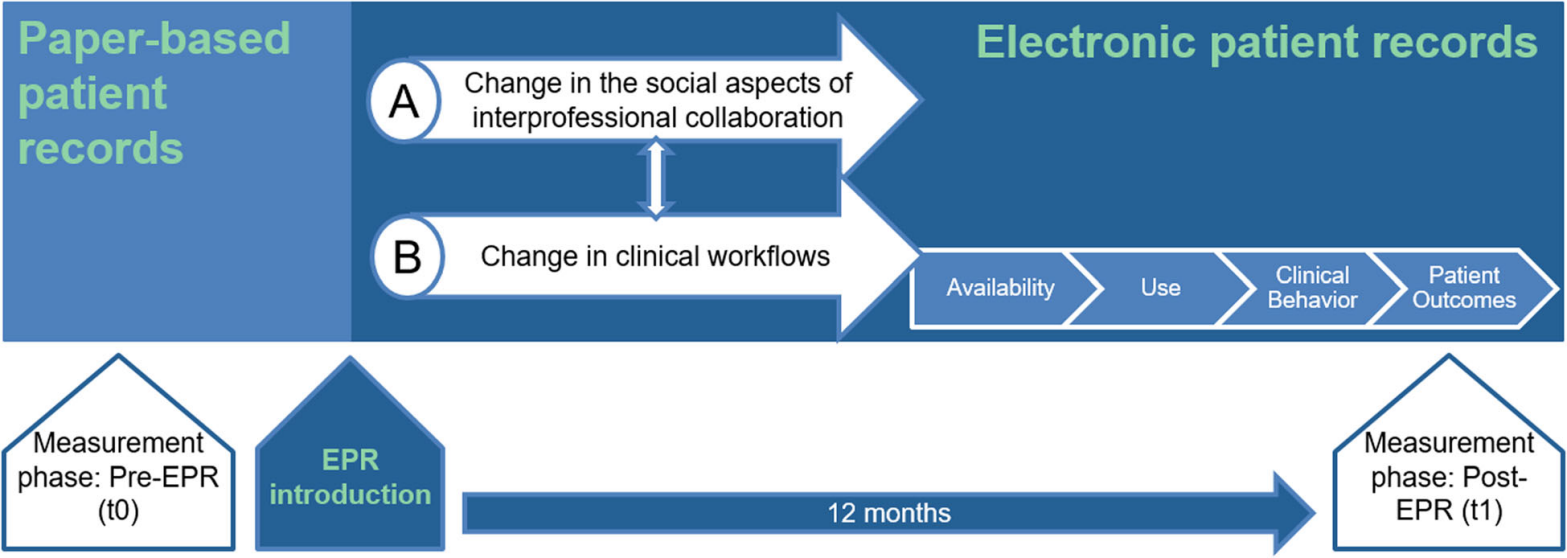
Study design for each hospital

The study *eCoCo* is a longitudinal case study integrating mixed methods from qualitative and quantitative social research, conducted from 05/2020 to 04/2023. Figure 1 shows the developed pre-post study design. All data are collected before the introduction of the EPR system (pre-EPR, t0) and again 12 months after its introduction (post-EPR, t1). Data are collected in three German hospitals, representing a multicenter case study. Recruitment of hospitals took place before the project started. Selected hospitals from North Rhine-Westphalia, Germany, were contacted by e-mail, informed about the study and asked about their interest in participating. Selection was conducted with the periodic sampling methods of Microsoft Excel. For hospitals that assented, the inclusion criteria were checked. Inclusion criteria comprised the introduction of EPR during the first half of the project duration. Exclusion criteria were working completely with EPR or having the EPR conversation not planned yet. Furthermore, the sample aimed to involve three urban hospitals of different sizes. After checking the inclusion and exclusion criteria, three of the eligible hospitals were randomly included, using the random sampling method of Microsoft Excel. To answer the research questions a formal meeting is conducted between the research team and relevant managers of each hospital to define a use case at the beginning of the study period. Those use cases form the examination unit for data collection and analysis and may encompass the total clinical workflow of a diagnosis or single processes such as ward rounds or medication processes.

The research of *eCoCo* is conducted by using multiple forms of qualitative and quantitative data collection in a convergent design to maximize the strengths and minimize the weaknesses of each type of data [45]. Table 1 provides an overview of the versatile methods used.

**Table 1:** Overview of methods and data in *eCoCo*

Data source	Research Question A			Research Question B		
	A1	A2	A3	B1	B2	B3
Participant observation	x	x	o	x		o
Interviews	x	x	o			o
Documents		o	x			o
Focus groups		o	o	x		o
Time measurement				x		o
Patient questionnaire					x	x
Employee questionnaire				o	x	x
Structural and administrative data						x
Questionnaire level of digitalization	o	o	o	o	o	x

Note. x = main data source for analysis; o = supplementary data source

## Research Question A: Methods and Analyses

To answer the first research question of if, how and why the introduction of EPR leads to changes in social aspects of interprofessional collaboration, the study integrates several methods of qualitative data collection and analysis. All collected qualitative data are analyzed individually and then triangulated. Triangulation of different data sources and combination of various analysis methods produces higher quality results [46, 47]. Furthermore, triangulation should better reveal communication improvements and challenges and allow deeper insights into changes in interprofessional collaboration.

### 1. Qualitative Network analysis

A qualitative network analysis is performed to answer research question A1. To perform a qualitative network analysis [48, 49], data from participant observations and interviews are used. The aim of **participant observations** is to observe different members of the professional groups involved in the use case and to investigate the reality of their everyday processes and practices without giving them the opportunity to adjust their behaviors [50]. In this way, the factors of interprofessional collaboration within patient care are focused. Each participant observation is conducted by shadowing one healthcare professional. The researcher keeps detailed field notes, records observations and documents conversations immediately after occurrence to ensure all details for analysis are collected [51]. Subsequently, the field notes are transferred into digital observation protocols and entered in MAXQDA for analysis. Following Hammersly and Atkinson [52], the three dimensions of context, people and time are considered for sampling. Shadowing ensures that healthcare professionals are observed in different contexts (e.g., in contact with patients and in interprofessional situations). Observations include nurses, physicians and, depending on use case, other professional groups, such as service personnel or administrative employees. Observations are repeated on working days. The study aims to complete this purposeful sampling in four to six weeks (two measurement phases: pre-EPR and post-EPR).

In the first analysis, qualitative content analysis [47] is used to extract the actors, interprofessional collaboration, and used communication channels (e.g., face-to-face conversation, telephone, e-mail) as categories from the observation protocols. To visualize how professional collaboration takes place in the observed ward, the categories are entered as actors and relational attributes into the VennMaker software tool [53]. The social network maps thus created are ego-centered, or from the perspective of the respective observed professional [54].

To expand and validate the social network maps, interviews with healthcare professionals are conducted at workplace. Interviews are carried out face-to-face or online with healthcare professionals affected by the introduction of EPR and involved in the selected use cases. Employees have to be over 18 years old and speak German. The interviews are recorded, transcribed and entered in MAXQDA.

For both, observations and interviews, the collected data is analyzed in an iterative process. If no more information about the use case, focusing on actors and communication channels, are added to the social network maps, the data collection is completed because data saturation has been reached. The visualized social network maps are analyzed with a focus on interprofessional collaboration and the handling of patient records. Social network maps are created both before and after EPR introduction to allow comparison of changes.

### 2. Reconstructive hermeneutic analysis

The second analysis is conducted to obtain insight into employees’ perspectives (research question A2). For the analysis, observational data are complemented with the interview and focus group data (see Sect. 4 Workflow analysis). All data are analyzed using a reconstructive hermeneutic approach [55]. Situations of joint actions (e.g., ward rounds) and handling of patient records are analyzed to determine formal and informal expectations, rules and norms and how these influence communication and interprofessional collaboration.

### 3. Document analysis

To understand the content and quality of patient records and potential changes because of the introduction of the EPR (research question A3), a **document** analysis [56] is conducted. For this purpose, all patient records including the use case originated the measurement phase are selected, anonymized and provided by each hospital. This is expected to result in a low, two-digit number of records (e.g., 15 to 30 records ) per measurement phase and hospital. Data saturation is reached by the complete data collection. Depending on the characteristics of the documentation, the records are analyzed by content [57], with a focus on various criteria for the quality of the documentation derived from existing literature, such as statements on the completeness of information or the levels of accuracy or compactness [58–60]. Conclusions about changes in documentation are drawn by comparing paper-based records with EPR. To interpret the results of the document analysis regarding communication, these results are triangulated with the results of the observations, interviews and focus groups.

## Research Question B: Methods and Analyses

The second part of the study aims to gain greater insight into changes in clinical workflows resulting from the introduction of the EPR. To answer the second research question, different quantitative and qualitative data sources are used (see Table 1) and combined for three different analyses.

### 4. Workflow analysis

To understand and analyze the workflow (research question B1) of the use case in each hospital, a focus group and time measurement is used to collect data. At the beginning of each time point, a focus group is conducted to map the process of the selected use case. The interprofessional discussion [61, 62] helps to develop a holistic point of view. Depending on the defined use case, around five employees of the respective professions (e.g., nurses, physicians, administrators) participate in the focus group. Employees are included if they work in the ward where EPR is introduced, belong to one of the professional groups involved in the use case, are over 18 years old, and speak German. The individual participants are instructed to explain the steps of the process. The discussions are recorded and transcribed. The described workflow is photographed in order to visualize it digitally as a detailed process map with Microsoft Visio. Participant observation data is used to further complete the process map.

Time measurements are conducted of the clinical workflow or respective process steps. Based on the previously constructed detailed process map, the amount of time healthcare professionals spend on activities like patient care or documentation is measured and documented. In addition to the duration of the process steps, the aim is to identify process-related consequences of changed tasks. To measure the time accurately, several approaches are applied depending on the use case: (1) following healthcare professionals by external observers with a stopwatch on selected days, (2) self-assessment of time by healthcare professionals, (3) estimation of time based on observation protocols, (4) estimation of selected tasks based on secondary data using timestamps of the EPR system, or (5) verification of estimated time via discussion rounds in interprofessional meetings.

### 5. Analysis of patients’ and employees’ assessment

To answer research question B2, patients treated in hospital wards where EPR is introduced receive a questionnaire. The questionnaire contains standardized scales to measure outcomes as per the CAMM (e.g., patient safety) as well as patients’ satisfaction and experience with the clinical workflows and communication processes. The questionnaire is supplemented with for the study developed scales on attitudes regarding the digitization of patient data and the impact of the COVID-19 pandemic to account for the current situation. In addition, further questions record patients’ personal characteristics (e.g., age, sex). Standards of questionnaire development are followed [63–65], and the questionnaire is subjected to cognitive pretesting. Assuming an effect size of 0.5, power of 0.8, and alpha of 0.05, a sample size of N = 64 patients is necessary to detect significant changes in patient assessment based on a power analysis. With a response rate of 90 % confirmed by previous studies [66], 72 patients per measurement phase within each hospital are recruited.

Secondly, the healthcare professionals are surveyed with an employee questionnaire that includes standardized scales to measure the provider dimensions of the CAMM (e.g., ethical, legal and safety concerns, job satisfaction), personal characteristics (e.g., age, profession) and potential influencing factors (e.g., expectations regarding EPR, affinity for technology, social capital). A for the study developed COVID-19 scale is added to be used as a control variable. The same methods for development and pretesting used in the patient questionnaire are applied for the employee questionnaire. For the sample size estimation, the same assumptions are made for the power analysis resulting in a required sample size of N = 34 employees to detect significant changes in employees’ assessment. Assuming a response rate of 50 %, as confirmed by previous studies [67, 68], 68 employees are recruited per measurement phase within each hospital.

The patient and employee surveys are conducted both online and on paper during the measurement phase of the observation. Patients and employees are included if they are treated in or working at the studied hospital ward, during the measurement phase, being over 18 years old and speaking German. The data are prepared with Teleform software (paper-based) and LimeSurvey (online) and analyzed with statistical programs (e.g., R, SPSS). Psychometric analyses are performed to ensure the reliability and validity of newly developed items and, if possible, scales. Previously validated scales are analyzed following the coding manuals. After descriptive analyses, inferential statistical analyses with the outcome variables between the pre-EPR and the post-EPR measurements are conducted to investigate potential changes resulting from EPR introduction.

### 6. Classification into CAMM

Finally, to classify the state of the ward into the CAMM (research question B3), structural and administrative data are collected for each hospital and each time of measurement, and the level of digitalization is measured. **Structural and administrative data** are provided by the hospital. Structural data are used to characterize the participating hospitals. Administrative data include, for example, length of stay or patient safety indicators and represent the patient outcome dimension of the CAMM. Thus, the use of administrative data allows to assess the pre- and post-EPR introduction outcomes and is analyzed by inferential statistics.

**Level of digitalization** is measured for each hospital pre- and post-EPR introduction. Since existing models offer a very broad grid that disregards regional specifics (e.g., the electronic medical report adoption model, EMRAM, [69]), a questionnaire is developed to provide detailed classification of digitalization level. The questionnaire includes two parts. The first part features questions about the system and implementation status of various system functions across the whole hospital and is conducted with the information technology department of each participating hospital. The second part assesses the technical functionality of the respective organizational unit and the availability and quality of patient data in the care process and is conducted with the medical management team of the organizational unit of the use case. The results of the questionnaire are used to describe the progress of digitalization from a technical perspective, which is considered in the interpretation of all results. Since the adoption of the EPR occurs step by step, it is important to include this perspective.

To be classified into the CAMM, all data collected for research question B are triangulated and combined with information from the data and analyses of research question A. This joint consideration of the quantitative and qualitative results and analyses allow classification of the state of adoption for each hospital in order to enhance understanding of workflow, outcomes and interprofessional collaboration.

## Discussion

The results of *eCoCo* will provide new understanding of the changes resulting from introducing EPR and thereby focusing on interprofessional collaboration and clinical workflows. The study uses a mixed methods design with a variety of qualitative and quantitative methods to benefit from a broad database and to account for the strengths and weaknesses of each method. Data are collected longitudinally before and after introducing EPR, which allows investigation of the direct consequences of the EPR introduction. The COVID-19 pandemic likely acts as a confounding factor and may also be an enhancing factor for digitalization in general, wherefore it is considered in this study. As far as the authors know, the study will be the first to mainly concentrate on the collaborative changes resulting of an EPR introduction focusing on employee’s perspective. Ethical and legal issues are considered as well as patient’s safety and patients’ perspectives on the quality of care to ensure its stability. The changes identified due to the introduction of EPR are relevant not only for the participating hospitals but for all hospitals, especially in light of the increasing spread of digitalization and increasing use of EPR. Assistance and support opportunities for health services will be developed at the end of the project and will be recorded in manuals and/or guidelines and disseminated by key persons.

Successful completion of the study requires consideration of challenges in data collection, analysis, and interpretation. To reduce limitations because of the anticipated challenges, the following actions are planned.

The quality of the results will depend largely on the willingness of sufficient healthcare professionals to participate in the various data collection methods. This target group has a high workload, wherefore suitable incentives for participation are provided and leaders are brought on board in advance. The questionnaire is kept as short as possible. Furthermore, as main data source the observation is used, which minimizes the effort for healthcare professionals.

To compare data of the pre- with the post-EPR measurement phase, theoretical considerations have to be made in advance. These considerations may result in restricted data collection if phenomena are overlooked. A constant balancing and reflection between structuring and openness towards the field are conducted during the measurement phases to prevent these restrictions. Balance between structure and openness should be achieved by constant member checking. This contains communicative validation with the healthcare professionals iteratively and thus leads to a verification of the findings.

The interpretation of qualitative results is highly vulnerable to subjectivity. Therefore, the research team includes advanced researchers trained in qualitative methods. Furthermore, the interprofessionality of the research group (including former healthcare professionals) takes into account the complexity of the research question. This is further complemented by including exchange opportunities with an advisory project board in all study phases. This project advisory board is constituted of experts in the relevant research fields (e.g., digitalization in healthcare, communication in healthcare, organizational change) and is founded to maximize scientific quality and topicality and to further disseminate the project aims and results.

This health services research study is composed of three case studies conducted in different hospitals with different use cases, which will make it possible to identify similarities and differences between them. However, the generalization of results will be limited in dependence of the study design and mainly qualitative methods used. Even though the results are restricted to a few hospital wards in Germany, the chance is to discuss the results of the analyses of both research questions to comprehensively explore the mechanisms and thereby the changes triggered by introducing EPR. *eCoCo* aims to gain deeper insights into EPR adoption overall in Germany and the mechanisms behind interprofessional collaboration in general. Therefore, this study provides a basis for future research.

## Data Availability

It is planned to submit the results of all analyses for publication in peer-reviewed journals and to present them at national and international conferences. The dissemination will also be supported by professional public relations activities. The respective datasets analyzed for publications will be included in the published articles. The anonymous datasets generated during the current study may be made available from the corresponding author on a reasonable request. Protocol modifications will be communicated to relevant parties such as the publisher of this study protocol or the trial registry.

## Abbreviations

eCoCo: Electronic Patient Record and its Effects on Social Aspects of Interprofessional Collaboration and Clinical Workflows in Hospitals
EPR: Electronic patient records
EPA: Elektronische Patientenakte
CAMM: Clinical adoption meta-model
IT: Information technology
pre-EPR: Before the introduction of electronic patient records
post-EPR: 12 months after the introduction of electronic patient records

## Footnotes

2 The term is not used consistently between health policy, healthcare professionals and literature in German and English. The study in hand uses ‘electronic patient records’ and ‘EPR’ [elektronische Patientenakte, EPA] to refer to the concept defined above. In the literature, the term ‘electronic medical records’ [elektronische Arzt-/Krankenakte] is often used interchangeably. These terms should not be confused with ‘electronic health records’ [elektronische Gesundheitsakte], which contain information about the medical history of a patient from all healthcare professionals involved in a patient’s care [5].
